# Seasonal variation in haematological and biochemical variables in free-ranging subadult brown bears (*Ursus arctos)* in Sweden

**DOI:** 10.1186/s12917-015-0615-2

**Published:** 2015-12-08

**Authors:** Anne Randi Græsli, Alina L. Evans, Åsa Fahlman, Mads F. Bertelsen, Stéphane Blanc, Jon M. Arnemo

**Affiliations:** Department of Forestry and Wildlife Management, Faculty of Applied Ecology and Agricultural Sciences, Hedmark University College, Campus Evenstad, NO-2418 Elverum, Norway; Center for Zoo and Wild Animal Health, Copenhagen Zoo, Roskildevej 38, DK-2000 Frederiksberg, Denmark; Department of Clinical Sciences, Faculty of Veterinary Medicine and Animal Science, Swedish University of Agricultural Sciences, P.O. Box 7054, SE-750 07 Uppsala, Sweden; Université de Strasbourg, IPHC, 23 rue Becquerel, 67087 Strasbourg, France; CNRS, UMR7178, 67087 Strasbourg, France; Department of Wildlife, Fish and Environmental Studies, Faculty of Forest Sciences, Swedish University of Agricultural Sciences, SE-901 83 Umeå, Sweden

**Keywords:** Brown bear, *Ursus arctos*, Haematology, Biochemistry, Seasonality

## Abstract

**Background:**

Free-ranging brown bears exhibit highly contrasting physiological states throughout the year. They hibernate 6 months of the year, experiencing a decrease in body temperature, heart rate, respiratory rate and metabolism. An increase in food consumption and the resulting weight gain (mostly through fat storage) prior to hibernation are also part of the brown bear’s annual cycle. Due to these physiological changes, haematological and biochemical variables vary dramatically throughout the year. Seasonal changes in 12 haematological and 34 biochemical variables were evaluated in blood samples collected from 40 free-ranging subadult brown bears (22 females, 18 males) immobilised in Sweden in winter (February-March), spring (April-May), and summer (June).

**Results:**

Higher levels of haemoglobin, haematocrit and red blood cell count, and a lower white blood cell count and mean cell volume was found during hibernation than in spring and summer. Lower values of the enzymes; aspartate aminotransferase (AST), alanine transaminase (ALT), alkaline phosphatase (AP), γ-glutamyl transpeptidase (GGT), glutamate dehydrogenase (GD) and amylase, and increased values of β-hydroxybutyrate (β-HBA) and blood lipids; triglycerides, cholesterol and free fatty acids, were present during hibernation compared to spring and summer.

**Conclusions:**

This study documents significant shifts in haematological and biochemical variables in samples collected from brown bears anaesthetised in winter (February-March) compared to in spring and summer (April-June), reflecting the lowered metabolic, renal and hepatic activity during hibernation. Lower values of enzymes and higher values of blood lipids during hibernation, likely reflect a lipid-based metabolism.

## Background

Hibernation is an important feature of the biology of the brown bear (*Ursus arctos*). Brown bears spend about half of the year in a den and survives extreme winter conditions without eating. Brown bears in Sweden enter their dens in October-November and emerge during March-April. The precise mechanisms determining den entry and exit are still unknown [1, 2]. Prior to hibernation, the bears increase their food consumption (primarily of berries) and store adipose tissue [3, 4]. During hibernation, their body weight is reduced by 20-40 %, depending on sex and age, and metabolism decreases with up to 75 % [5, 6], becoming mainly lipid based [7]. However, this decrease in metabolism is associated with only a moderate drop in body temperature. Body temperatures down to 32 °C during immobilisation in winter (February-March), have been documented in brown bears in Scandinavia [8]. Compared to resting heart rate in the active season, hibernating brown bears are bradycardic [3, 9]. A 35 % decrease in heart rate and a 69 % decrease in stroke volume during hibernation, compared to during summer, have been reported in subadult brown bears anaesthetised with a drug combination including medetomidine [9].

Blood variables are important in understanding the impact of disease on both an individual and a population level and to assess the health of individual animals [10]. Seasonal influences on blood variables of brown bears can also aid in understanding hibernation physiology. Several reports have demonstrated seasonal variations for selected haematological variables in brown bears [11–15]. This however, is the first multi-season report of changes in blood variables of hibernating free-ranging brown bears. The objective of this study was to describe seasonal changes in haematological and biochemical variables for free-ranging subadult brown bears in Sweden. Only subadult bears were included in this study, because female adult bears may have newborn cubs during hibernation and because it may be risky for the personnel to wake up male adult bears from hibernation. Variation between age categories have earlier been studied in brown bears [11, 14–16], because of these results we don’t expect a difference in subadult and adult bears, which means that the data could infer to adult bears.

## Methods

### Study site and animals

This study included 40 free-ranging subadult (2-4 years old) brown bears (22 females, 18 males), which were immobilised in the period of 2006 through 2013. Mean age at the time of immobilisation for females and males were 2.9 and 2.7 years old, respectively. Season was assigned to three categories according to the date the bear was immobilised; winter (immobilised from the 4^th^ of February to the 2^nd^ of March), spring (immobilised from April 12^th^ to May 3^rd^) and summer (immobilised between the 8^th^ to the 23^rd^ of June). The grouping was based on the seasonal changes in the bears’ life during the year, with winter being the hibernation period, spring the transition period and summer the active period. There was at least one month gap between each season group.

Blood samples from bears captured during spring (April-May) were included in a previous study [16]. The study area was in the county of Dalarna, Sweden, approximately 61°N, 15°E. Blood samples were collected during immobilisation for GPS collaring for ecological studies within the Scandinavian Brown Bear Research Project. All bears appeared clinically healthy as determined by clinical examination during immobilisation. All sampling was approved by the Ethical Committee on Animal Experiments, Uppsala, Sweden (application numbers: C 7/12, C 47/9 and C 59/6).

During spring and summer (April–June), bears were immobilised by remote darting from a helicopter using medetomidine-zolazepam-tiletamine, as previously described [17]. In February and early March, bears were immobilised in their winter dens [8]. Subadult bears, previously fitted with global positioning system (GPS) collars and very high frequency (VHF) abdominal implants, were selected and localised by radio tracking. Field workers transported the equipment with snowmobiles within 200-800 m of the den, and thereafter approached the den on skis. After removing the snow, a metal grate was placed over the entrance, and the bear was darted with medetomidine-zolazepam-tiletamin-ketamine [8].

### Sample collection and transport

Blood was sampled as reported previously [16] within 60 min of immobilisation from the jugular vein using a vacutainer system (BD Vacutainer®, BD Diagnostics, Preanalytical Systems, Franklin Lakes, NJ, USA). Blood for haematological analyses was collected in 4 mL tubes, with EDTA (ethylenediaminetetraacetic acid) as anticoagulant (Vacuette®, Greiner Bio-One International AG, Kremsmünster, Austria). The blood samples were kept cool from sampling until analysis at the Clinical Chemistry Laboratory at the Swedish University of Agricultural Sciences, Uppsala, Sweden. The time from sampling to analysis was within two days.

Blood for biochemistry was collected in 9 mL tubes with gel and clot activating factor (Vacuette®, Greiner Bio-One International AG). The samples were kept at room temperature for 1-2 h to ensure complete clotting, and then centrifuged at 1500 g for 10 min to separate the serum. The serum was stored in 2 mL cryogenic vials (Nalgene, Nalge Company, Rochester, NY, USA) and kept at -20 °C until shipment to the Central Laboratory, Norwegian School of Veterinary Science, Oslo, Norway. The samples were kept cool during shipment.

### Laboratory analyses

The haematological analyses and complete blood cell counts were carried out upon arrival at the laboratory. For haematological analyses in 2006 and 2007, a Cell-Dyn 3500 Hematology Analyser (Abbott Laboratories, Abbott Park, Illinois, USA) was used. In 2008, the Cell-Dyn 3500 was replaced by an ADVIA®2120 Hematology System (Bayer HealthCare, Diagnostics Division, Tarrytown, NY, USA). Cell differentiation was carried out by manual examination of a blood smear, by the same technician at the laboratory.

The haematology profile included red blood cell (RBC) count, white blood cell (WBC) count, haemoglobin, haematocrit, mean corpuscular volume (MCV) and mean corpuscular haemoglobin concentration (MCHC). In addition, a white blood cell differential count (percentage of total) for band shaped neutrophils, segmented neutrophils, eosinophils, basophils, lymphocytes and monocytes was included.

All clinical chemistry analyses, except cortisol analysis, were carried out using an ADVIA®1650 Chemistry System (Siemens Healthcare, Tarrytown, NY, USA) from 2006 to October 2011. Thereafter it was replaced with an ADVIA®1800 Chemistry System (Siemens Healthcare). The methods were the same for both analysers. Upon arrival at the Central Laboratory, samples were kept at -80 °C until analysis. Cortisol was measured with an Immulite system (Diagnostic Products Corporation, Los Angeles, CA, USA). The protein fractions were measured by Paragon CZE TM 2000 Capillary Electrophoresis System (Beckman Coulter, Brea, California, USA) from 2006 to September 2008, thereafter it was replaced with a Capillarys TM 2 (Sebia®, Evry, France).

The biochemical profile included aspartate aminotransferase (AST), alanine transaminase (ALT), alkaline phosphatase (AP), γ-glutamyl transpeptidase (GGT), glutamate dehydrogenase (GD), creatinine kinase (CK), amylase, lipase, lactate dehydrogenase (LD), albumin, α_1_-globulin, α_2_-globulin, β-globulin, γ-globulin, albumin-globulin ratio, total protein, β-hydroxybutyrate (β-HBA), bile acid, total bilirubin, cholesterol, creatinine, free fatty acids (FFA), glucose, triglycerides, urea, uric acid, calcium, chloride, iron, magnesium, inorganic phosphorous, potassium, sodium, and cortisol. Not all analyses were performed on each bear.

### Statistical analysis

JMP®10.0.0 statistical software (SAS Institute, Cary, North Carolina, USA), was used to perform statistical analysis, and a *p*-value of <0.05 was considered significant. Eight bears were resampled in the same season during multiple years; in these cases, one sample from each bear in each season was randomly selected using a random number generator. The population was characterised based on sex (male and female) and the season during which the blood was collected.

The distributions of the haematological and biochemical variables were tested for normality. Those with an approximate Normal distribution were kept on the direct scale, whereas non-normal variables were transformed to normality with a Box-Cox transformation.

The formula for the Box-Cox transformation, where $\overline{y}$ is the geometric mean [18];

$$ y{\hbox{'}}_{\uplambda}=\frac{y^{\uplambda}-1}{\uplambda \cdot {\overline{y}}^{\uplambda -1}},for\uplambda \ne 0,\kern0.5em \mathrm{and}\kern0.5em y{\hbox{'}}_{\uplambda}=\overline{y}\kern0.5em \cdot \log \cdot (y),for\uplambda =0 $$

When >90 % of the values for a given variable was 0, transformation was considered non-sensible, therefore the reference values for these variables were presented as 90^th^, 97.5^th^ and 100^th^ percentiles. For variables including observed values of 0, a factor of 0.05 or 0.5 was added to all values before transformation.

Comparisons between sex and seasons were performed on the transformed variables using analysis of variance. A significant *F*-test was followed by a simultaneous comparison of paired means using the Tukey-Kramer test. After calculating the 95 % confidence intervals in the significantly different seasons, the transformed data were reconverted to the original data scale and corrections were made for any added values. For variables for which significant differences were not detected, sex and season cohorts were grouped together for presentation. Data is presented as 95 % confidence intervals.

## Results

Results for haematological and biochemical variables for subadult bears in different seasons are presented in Tables 1, 2, 3, 4 and 5. The λ-values from the Box-Cox transformations are presented.

**Table 1:** Seasonal changes in haematological variables for subadult brown bears (*Ursus arctos*) presented as 95 % confidence interval

Variable^a^	Unit	λ^b^	2.5 - 97.5 interval^c^	Sex^d^	Season^e^	*n*	Range	*p*-value^f^
Haemoglobin	g/L	2	141-213	F + M	W	11	187–215	<0.0001
					Sp	19	153–187	
					Su	15	134–183	
RBC	×10^12^/L	0.8	6.1–9.5	F + M	W	11	8.4–10.0	<0.0001
					Sp + Su	34	6.2–8.0	
Haematocrit	L/L	−0.2	0.40–0.63	F + M	W	11	0.54–0.66	<0.0001
					Sp	19	0.46–0.55	
					Su	15	0.42–0.53	
MCV	fL	−0.6	60–75	F + M	W	11	58–71	0.0038
					Sp + Su	34	62–74	
MCHC	g/L	2	289–385	F + M	W + Sp + Su	45	318–388	
WBC	×10^9^/L	0	2.7–13.2	F + M	W	11	3.1–12.7	0.0208
					Sp + Su	34	3.9–18.3	
Segm.neutroph.	%	2	41.3–89.5	F + M	W	11	31.4–70.8	<0.0001
					Sp + Su	34	50.7–87.9	
Eosinophils	%	0.4	0.0–21.4	F + M	W	11	1.2–18.4	0.0091
					Sp + Su	34	0.0–10.7	
Lymphocytes	%	0.6	2.6–44.3	F + M	W	11	15.1–45.4	0.0004
					Sp + Su	34	3.1–34.9	
Monocytes	%	0.4	1.0–21.4	F + M	W + Sp + Su	45	0.6–14.6	

^a^RBC = red blood cell count; MCV = mean corpuscular volume; MCHC = mean corpuscular haemoglobin concentration; WBC = white blood cell count; Segm.neutroph. = segmented neutrophils

^b^λ = the transformation value from the Box-Cox transformation

^c^2.5 and 97.5 percentiles on untransformed data

^d^F = female; M = male. ^e^:W = winter (February-March); Sp = spring (April-May); Su = summer (June)

^f^ *p*-values are given for significantly different seasons groups

**Table 2:** Seasonal changes in serum enzymes for subadult brown bears (*Ursus arctos*) presented as 95 % confidence interval

Variable^a^	Unit	λ^b^	2.5 - 97.5 interval^c^	Sex^d^	Season^e^	*n*	Range	*p*-value^f^
AST	U/L	−1.2	43–317	F + M	W	15	38–95	<0.0001
					Sp	27	46–114	
					Su	18	46–437^g^	
ALT	U/L	−1	11–63	F + M	W	15	10–20	<0.0001
					Sp	27	12–33	
					Su	18	11–76^g^	
AP	U/L	−0.4	13–214	F + M	W	15	12–35	<0.0001
					Sp	27	33–111	
					Su	18	27–221^g^	
GGT	U/L	−0.4	4–98	F + M	W	15	4–16	<0.0001
					Sp + Su	45	6–61	
GD	U/L	−0.2	2–24	F + M	W + Sp	42	2–7	0.009
					Su	18	1–33	
CK	U/L	−0.6	56–843	F + M	W + Sp + Su	60	61–522	
Amylase	U/L	−0.4	26–207	F + M	W	15	23–86	<0.0001
					Sp + Su	45	36–168	
Lipase	U/L	−1.2	12–150	F	W + Sp + Su	33	15–197^g^	0.0018^S^
				M	W + Sp + Su	27	11–43	
LD	U/L	0.6	379–1021	F + M	W	15	363–753	<0.0001
					Sp	27	465–928	
					Su	18	565–1054	

^a^AST = aspartate aminotransferase; ALT = alanine transaminase; AP = alkaline phosphatase; GGT = γ-glutamyl transpeptidase; GD = glutamate dehydrogenase; CK = creatinine kinase; LD = lactate dehydrogenase

^b^λ = the transformation value from the Box-Cox transformation

^c^2.5 and 97.5 percentiles on untransformed data

^d^F = female; M = male

^e^W = winter (February-March); Sp = spring (April-May); Su = summer (June)

^f^ *p*-values are given for significantly different seasons groups: S = *p*-value for sex

^g^Non-estimable 95 % confidence interval, the range is given as lowest-highest measured value

**Table 3:** Seasonal changes in serum proteins for subadult brown bears (*Ursus arctos*) presented as 95 % confidence interval

Variable^a^	Unit	λ^b^	2.5 - 97.5 interval^c^	Sex^d^	Season^e^	*n*	Range	*p*-value^f^
Albumin	g/L	1	30.6–54.4	F + M	W	15	41.6–55.6	<0.0001
					Sp	27	34.0–49.0	
					Su	18	28.3–47.0	
α_1_-globulin	g/L	−1.2	2.7–7.4	F + M	W + Sp + Su	60	2.6–7.5	
α_2_-globulin	g/L	0	2.9–7.4	F + M	W	15	4.5–7.9	<0.0001
					Sp + Su	45	2.8–6.6	
β-globulin	g/L	−0.4	5.2–12.5	F	W	8	5.4–11.6	<0.0001, 0.0009^s^
					Sp	15	3.3–9.5	
					Su	10	5.4–11.6	
				M	W	7	6.1–12.2	
					Sp	12	5.5–9.8	
					Su	8	6.1–12.2	
γ-globulins	g/L	0.6	2.4–6.4	F + M	W + Sp + Su	60	1.3–2.2	
A:G		0	1.41–3.04	F + M	W + Sp	42	1.55–3.04	0.0022
					Su	18	1.32–2.59	
Total protein	g/L	0.8	51–77	F + M	W	15	64–79	<0.0001
					Sp + Su	45	49–70	

^a^A:G = Albumin:globulin ratio

^b^λ = the transformation value from the Box-Cox transformation

^c^2.5 and 97.5 percentiles on untransformed data

^d^F = female; M = male

^e^W = winter (February-March); Sp = spring (April-May); Su = summer (June)

^f^ *p*-values are given for significantly different seasons groups: S = *p*-value for sex

**Table 4:** Seasonal changes in serum metabolites for subadult brown bears (*Ursus arctos*) presented as 95 % confidence interval

Variable^a^	Unit	λ^b^	2.5 - 97.5 interval^c^	Sex ^d^	Season^e^	*n*	Range	*p*-value^f^
β-HBA	mmol/L	0.2	0.0–1.2	F + M	W	15	0.5–1.4	<0.0001
					Sp	27	0.0–0.8	
					Su	18	0.0–0.2	
Bile acids	μmol/L	−1	4–52	F + M	W	15	4–9	<0.0001
					Sp + Su	45	6–36	
Bilirubin (total)	μmol/L	0.2	0–4	F + M	W + Sp	42	0–4	0.0131
					Su	18	0–2	
Cholesterol	mmol/L	1	4.3–12.6	F + M	W	15	8.7–12.8	<0.0001
					Sp + Su	45	4.0–11.4	
Creatinine	μmol/L	0.2	72–303	F + M	W	15	166–322	<0.0001
					Sp	27	105–196	
					Su	18	52–152	
FFA	mmol/L	0.4	0.1–1.1	F + M	W + Sp	42	0.2–1.2	<0.0001
					Su	18	0.1–0.8	
Glucose	mmol/L	0.8	2.6–10.6	F + M	W + Sp	42	4.0–10.2	0.0003
					Su	18	1.9–9.2	
Triglycerides	mmol/L	0.4	1.8–7.2	F + M	W	15	3.4–7.8	<0.0001
					Sp	27	2.1–5.3	
					Su	18	1.7–4.1	
Urea	mmol/L	0	0.5–26.3	F + M	W	15	1.0–12.1	<0.0001
					Sp	27	0.3–9.5	
					Su	18	0.6–32.4^g^	
Uric acid	μmol/L	−0.6	48–270	F + M	W	15	42–146	<0.0001
					Sp + Su	45	68–215	

^a^β-HBA = β-hydroxybutyrate; FFA = free fatty acids

^b^λ = the transformation value from the Box-Cox transformation

^c^2.5 and 97.5 percentiles on untransformed data

^d^F = female; M = male

^e^W = winter (February-March); Sp = spring (April-May); Su = summer (June)

^f^ *p*-values are given for significantly different seasons groups

^g^Non-estimable 95 % confidence interval, the range is given as lowest-highest measured value

**Table 5:** Seasonal changes in serum minerals and cortisol for subadult brown bears (*Ursus arctos*) presented as 95 % confidence interval

Variable	Unit	λ^a^	2.5 - 97.5 interval^b^	Sex^c^	Season^d^	*n*	Range	*p*-value^e^
Calcium	mmol/L	2	1.9–2.6	F + M	W + Sp + Su	60	1.9–2.6	
Chloride	mmol/L	2	92–103	F + M	W + Sp	42	92–102	0.0006
					Su	18	95–105	
Iron	μmol/L	0.2	12–48	F + M	W	15	12–26	<0.0001
					Sp + Su	45	16–51	
Magnesium	mmol/L	1.6	0.71–1.25	F + M	W	15	0.98–1.30	<0.0001
					Sp + Su	45	0.67–1.08	
Phosphorous (inorganic)	mmol/L	1.2	0.8–2.4	F + M	W + Sp	42	0.8–2.3	<0.0001
					Su	18	1.2–2.8	
Potassium	mmol/L	−1.8	3.4–5.6	F + M	W + Sp + Su	60	3.5–5.5	
Sodium	mmol/L	2	130–143	F + M	W + Su	33	133–142	0.0003
					Sp	27	129–141	
Cortisol	nmol/L	0.6	29–734	F + M	W + Sp + Su	57	25–668	

^a^: λ = the transformation value from the Box-Cox transformation

^b^ :2.5 and 97.5 percentiles on untransformed data

^c^:F = female; M = male

^d^:W = winter (February-March); Sp = spring (April-May); Su = summer (June)

^e^ : *p*-values are given for significantly different seasons groups

For the variables band-shaped neutrophils and basophils >90 % of the values were 0, they were presented as 90^th^, 97.5^th^ and 100^th^ percentiles. The percentiles for band-shaped neutrophils and basophils were 2.5; 11.3 and 12.4 and 0.0; 1.3 and 1.4, respectively.

Seasonal changes were found in eight out of twelve haematological variables and in all biochemical variables except creatinine kinase, calcium, potassium, α_1_-globulin, γ-globulins, lipase and cortisol. Haemoglobin, haematocrit, albumin, β-hydroxybutyrate, creatinine and triglycerides were significantly higher during winter compared to spring, and significantly higher during spring compared to summer. For the serum enzymes alanine transaminase, aspartate transaminase, alkaline phosphatase and lactate dehydrogenase the values during summer were significantly higher than during spring, and the values during spring were significantly higher than during winter. The values of red blood cell count, eosinophils, lymphocytes, total protein and cholesterol were significantly higher during winter compared to spring and summer, while the values of mean corpuscular volume, white blood cell count, segmented neutrophils, amylase, γ-glutamyl transpeptidase and bile acids were significantly lower during winter compared to spring and summer.

## Discussion

Uniquely, this study documents seasonal differences in blood variables in free-ranging brown bears. Bears were captured in their natural habitat and, when hibernating, inside their dens. Previous studies have used limited numbers of captive bears or non-hibernating bears. The seasonal changes in blood variables demonstrated here reflect the drastic physiological changes that bears undergo during the year. From the hibernation period in winter with decreased metabolism and reduced renal, liver and pancreas function, to the active state during summer where bears increase their food consumption to prepare for hibernation.

During the capture event there are several factors that potentially can affect the blood variables; both the immobilising drugs, and the capture method chosen to enable darting may affect the animals’ physiology [17, 19]. In the present study the bears in April-June were immobilised from a helicopter using a combination medetomidine-zolazepam-tiletamine. In February-March, when hibernating, ketamine was added to the drug combination, and the bears were immobilised inside the den.

High amounts of haemoglobin, red blood cells and haematocrit in winter have also been reported in other bear studies [11, 12, 20–22] and, as accompanied by higher total protein and albumin, can likely be attributed to dehydration and haemoconcentration. Higher albumin levels during hibernation compared to the other two seasons, and a nearly constant level of total globulins throughout the three seasons, gave a rise in the albumin:globulin ratio during winter. The lower mean cell volume in winter compared to in spring and summer, has also been reported in black bears [23] and is possibly related to iron status. Indeed, iron levels found in this study were significantly lower during winter than in summer and spring.

Several reports, including this present study, have shown lower white blood cell counts in bears during hibernation [12, 13, 20, 24]. One previous study in brown bears has reported lymphocytosis during winter [12]. Neutropenia during winter has been previously reported for brown bears [24]. This may, in addition to the decrease in total white blood cell count, be a consequence of a suppressed innate immune system during hibernation [24].

Lower enzyme levels during winter, as demonstrated in this study, have been reported in black bears [23, 25, 26]. Long-term fasting involves three phases based on changes in body tissue utilization as energy sources [27]. Fasting in bears was found to follow phase II [25] with increased fat catabolism associated with use of ketone bodies as substitute substrates for glucose. This inhibits the gluconeic pathway and reduces the membrane transport of glucose, and as a result of this, the demand for protein breakdown in the body is decreased [25, 27]. Alanine transaminase, aspartate transaminase, lactate dehydrogenase, γ-glutamyl transpeptidase, glutamate dehydrogenase and amylase are all enzymes that catalyse reactions involved in amino acid deamination, anaerobic glycolysis, glutathione reaction, conversion of glutamate to 2-oxoglutarate and hydrolysis of starches [28]. The decrease in protein breakdown and inhibition of the gluconeic pathway during hibernation may explain the significantly lower enzyme levels during winter. The lower values in 5 out of 9 enzymes during spring compared to summer, may be a reflection of a relatively slow increase in metabolic rate following den emergence [6]. A decrease in alkaline phosphatase during winter has been reported earlier in black bears [23], and may reflect decreased bone turnover.

Creatinine and urea are good indicators of renal function, with high values indicating impaired function [28]. During hibernation the glomerular filtrations rate (GFR) in bears decreases by about 70 % [29]. As a result, a rise in creatinine and magnesium concentrations in the blood is expected, and as far as creatinine goes, this has been previously documented in hibernating brown and black bears [12, 23, 30]. An increase in urea concentration is also expected, because of the decreased GFR, however, this study along with several previous reports [12, 23, 31] demonstrated a decrease. In a study on starvation of black bears during summer, bears became both dehydrated and azotemic [31]. The reason for decreased urea concentrations during hibernation is that the urea formed during catabolism of proteins is hydrolysed in the intestinal tract, and the nitrogen formed re-enters the protein synthetic pathways. This process is faster than the synthesis of urea [31, 32]. Hibernating bears are mainly metabolising fat, and their urea production decreases partly due to the shift from protein to fat metabolism. It has also been shown that the ability of bears to recycle urea during hibernation is not associated with den entry and exit, which means that it can occur before denning and some days after the bear has left the den [33]. This can explain the decreased urea values during spring compared to winter in this study, since the bears were captured shortly after leaving the den.

Increased values of blood lipids; triglycerides, cholesterol and free fatty acids, during hibernation found in this study, have been reported earlier in bears [22, 23, 25, 26, 34–36]. Catabolism of fat supplies the energy for metabolism during hibernation and the increase of blood lipids during hibernation, reflects lipolysis from adipose tissue [26, 37]. Bile acids are released during digestion, this leads to a postprandial increase in concentration of bile acids in the blood. The lower values of bile acids found in the present study, during winter, are consistent with fasting [28].

β-hydroxybutyrate (β-HBA) was also significantly higher in the winter compared to the spring and summer; as previously reported for denning bears [26, 32]. Even though β-HBA levels rise during hibernation, they are not high enough to result in ketosis [32]. In a study of changes in hepatic gene expression during hibernation, results indicated that the prevention of ketosis in hibernating bears is achieved by a rapid consumption of ketone bodies by peripheral tissue and not by a reduction of ketogenesis [26]. This process results in glucose sparing, likely an advantage for the fasting bear.

The changes reported here represent the many aspects of altered physiology seen during winter. The increased haemoglobin, red blood cells, haematocrit, total protein and albumin can be attributed to hypovolemia from the lack of liquid intake. The shift from carbohydrate and protein metabolism to fat metabolism results in decreased urea production and reduced demand for the enzymes important for protein breakdown (alanine transaminase, aspartate transaminase, lactate dehydrogenase, γ-glutamyl transpeptidase, glutamate dehydrogenase and amylase). Fat catabolism also resulted in increased blood lipids, triglycerides, cholesterol and free fatty acids and fasting resulted in increased β-HBA, lower bile acids, enzyme and urea levels. The general depression of metabolic rate is also represented by the decreased organ function including decreased kidney (increased creatinine and magnesium), liver (increased ALP), and pancreas (decreased amylase) function and suppression of the innate immune system in winter (neutropenia).

## Conclusion

Significant seasonal differences in hematological and biochemical variables were documented in free-ranging subadult bears. The changes show the shifts in activity of the liver, kidneys, pancreas and overall metabolic processes that characterizes long term fasting and hypometabolism.
